# Supplementary material for: Chromothripsis during telomere crisis is independent of NHEJ, and consistent with a replicative origin
Source: Genome Res. 2019 May;29(5):737–49. doi: 10.1101/gr.240705.118 (PMC6499312; doi:10.1101/gr.240705.118)
Supplement: Supplemental Material [file supp_gr.240705.118_Supplemental_file_1.zip › contigs/annotated_contigs/DB73/contig.2.DB73_length_609_mean_cov_13.6962233169.docx]

**DB73_length_609_mean_cov_13.6962233169**

```
CCTGGGATCTCGCCACACCAAAAATTAGATTATGGTCCTCTTCAGCCTGAAGAGACTCACCATGGCTTAAGAAGAAACAAACCTCAGAA
 >chr7:75976072-75976347 + E=9e-154
GGCCCTTCGTGAACTGTTTCCTGTTAACCTCTTTCAACTCATTTCCTGCTACTCCCTCAAAAAGCGCACCACCACAGCATTCCCTGAAT

GCCACCAACAAGGACTAAACAAATCACGCCACCAGTTCTCCTTTTCCATCCTGAAACTAAGCGTCCTATGCCCTCTGCCTGGCTATTCC

TTCCTC|CA|CCATGCCCAGCTAATTTTTGTATTTTTAGTAGAGATGGGGTTTCACCATATTGGCCAGTCTAGTCTTGAACTCCTGACC
       >chr7:75968328-75968653 - E=1e-173
TCAGGTGATCCGCCCACCTTGGCCTCCCAAAGTGGGAGGATTACACTGGGATTACAGGCGTGAGCCACCGCGCCCAGCCTATTACCATT

ATTTTTATACAACTAAGTTAAAACACACAGAAAGCAAAGAAGTGTGGTTTGAGTTTGTTGTATACATCCATAAACTGTTTATATAAAAT

TACTGGCTTTGGGCTTTTCTGAAAACTTGGCTTTCAAGATTGGCTTTAAGCTTCAATTGTAGGTATGAATAACAGGG
```
